# Investigation of the Role of Copper Species-Modified Active Carbon by Low-Temperature Roasting on the Improvement of Arsine Adsorption

**DOI:** 10.1021/acsomega.2c01355

**Published:** 2022-05-11

**Authors:** Xiaoyu Chen, Xuan Feng, Yibing Xie, Langlang Wang, Lu Chen, Xueqian Wang, Yixing Ma, Ping Ning, Yu Pu

**Affiliations:** † Faculty of Environmental Science and Engineering, Kunming University of Science and Technology, No. 727, Jingming South Road, Chenggong New District, Kunming 650500, China; ‡ Zhejiang Nanhua Anti-corrosion Equipment Co., LTD., Hangzhou 311255, China; § Faculty of Business Management, Yunnan Communications Vocational and Technical College, Kunming 650500, China

## Abstract

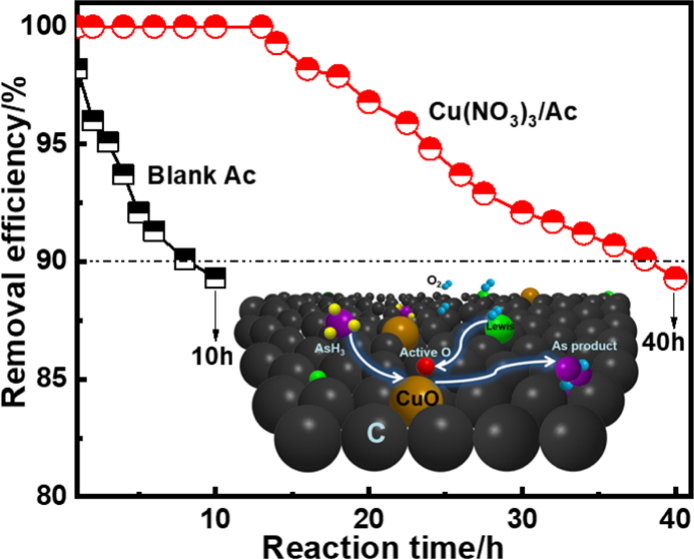

Traditional adsorbents undershot the expectations for arsine (AsH_3_) removal under low-temperature operation conditions in the industry. In this study, the copper (Cu) precursor was used to modify activated carbon and yield novel adsorbents by low-temperature roasting for high-efficiency removal of AsH_3_. The best conditions were determined as impregnation with 2 mol/L Cu(NO_3_)_2_ adsorbent and roasting at 180 °C. At a reaction temperature of 40 °C and an oxygen content of 1%, the AsH_3_ removal efficiency reached over 90% and lasted for 40 h and the best capacity of 369.6 mg/g was obtained with the Cu/Ac adsorbent. The characterization results showed the decomposition of Cu(NO_3_)_2_ during the low-temperature roasting process to form surface functional groups. The formation of the important intermediate Cu_2_(NO_3_)(OH)_3_ in the decomposition of Cu(NO_3_)_2_ into CuO plays a role in the good regeneration performance of the Cu/Ac adsorbent using water washing and the gas regeneration method. The results of in situ diffuse reflectance infrared Fourier transform spectroscopy combined with X-ray photoelectron spectroscopy demonstrated that the interaction of trace oxygen with Lewis (L) acid sites increased chemisorbed oxygen by 17.34%, significantly promoting the spontaneity of AsH_3_ oxidation reaction. These results provide a friendly economic method with industrial processes practical for AsH_3_ removal.

## 1 Introduction

The industrial sources of AsH_3_ mainly originate from phosphorous, coal, non-ferrous metal smelting, petroleum processing, and refining industries.^1^ AsH_3_ is a severe hemolytic poison that leads to the accumulation of hydrogen peroxide and destruction of the cell membrane. Acute AsH_3_ poisoning is characterized by acute intravascular hemolysis and renal damage. This may also involve and damage many important organs, including the heart, liver, and lungs.^2^ Meanwhile, AsH_3_ is a pollutant that causes adsorbent poisoning during carbonyl transformation and the synthesis of adsorbents.^3^ During the combustion process of arsenic in the yellow phosphorus ore, AsH_3_ can easily be produced by side reactions with newly formed H_2_. However, the low-temperature catalytic processes of such adsorbents could significantly lower the operating energy costs and reduce CO_2_ production for thermal oxidation. After high-pressure water washing, the exhaust gas temperature would drop to 30–50 °C. Hence, removing AsH_3_ without reheating may reduce heating energy consumption. Therefore, it is of practical significance and economic value to study adsorbents with high removal efficiencies of AsH_3_ under low-temperature conditions.

Activated carbon through modification has long been studied as an adsorbent carrier due to its low cost, widely available sources, resistance to acids and alkalis, excellent adsorption performances, large specific surface area, and non-toxicity. To this end, various modification methods have been attempted. For instance, Xu et al.^4^ carried out a redox pretreatment in Cu-modified activated carbon to increase the dispersion of Cu for CO removal. Islam et al.^5^ successfully prepared activated carbon from coconut shells and recorded a specific surface area of 1640 m^2^/g with a pore volume of 1.032 cm^3^/g. Khuong et al.^6^ prepared activated carbon adsorbents by K_2_CO_3_ activation for CO_2_ capture at room temperature. However, the activated carbon parameters can be modified by physical and chemical treatments to adjust and adapt the adsorbents to a wider range of applications, such as the removal of AsH_3_. Besides, activated carbon from commercial coal-based activated carbon possesses a very high affinity to arsenic and thereby may suit removal of AsH_3_ issued from industrial waste gas.

Several modification routes of activated carbon currently exist. Cu is the most suitable component for the removal of AsH_3_ by activated carbon due to its low heat release during the redox process coupled with a good removal effect on AsH_3_. As a result, numerous materials based on Cu have been used as adsorbents for the effective removal of AsH_3_. However, high operating temperatures are typically required in these processes.^7^ For instance, Lin et al.^8^ determined the optimal parameters of AsH_3_ removal by CuZnAl hydrotalcite-like adsorbents with calcination at 500 °C and a hydrothermal temperature at 105 °C. Uffalussy et al.^9^ performed adsorption of AsH_3_ by the Cu–Pd alloy using a high-throughput composition spread alloy film sample library. Jiang et al.^10^ noticed that a reaction temperature of 60 °C and an oxygen content of 4% allowed Co/Cu/Ac to effectively adsorb AsH_3_ after calcination at 400 °C with the adsorption capacity of AsH_3_ reaching 35.7 mg/g. Wang et al.^11^ reported adsorbents with 0.2 mol/L Cu(NO_3_)_2_ after calcination at 400 °C to possess superior activity towards the removal of AsH_3_. Besides, a high capacity of 43.7 mg/g adsorbent at 60 °C and 1.0% oxygen were obtained with Cu/Hβ. Previous studies by Huang^12^ showed a reduction in reaction temperature but still required higher calcination temperatures with stringent requirements on adsorbent preparation conditions. Alternatively, roasting is easier than calcination and suitable for practical applications.

In this study, a series of copper-modified activated carbon synthesized by roasting at low temperatures (80–180 °C) were investigated to gain a better understanding on the mechanism of CuO action for AsH_3_ removal. To this end, adsorbents with different precursors (Cu(NO_3_)_2_, CuCl_2_, Cu(C_2_H_4_O_2_)_2_, CuSO_4_) were prepared and AsH_3_ removal was tested under the actual low temperature in the industry. The preparation conditions related to loading precursor, loading concentration, roasting temperature, and preparation conditions linked to reaction temperature and oxygen content were all studied to determine the optimal conditions. The physico-chemical properties, phase compositions, and surface properties of the as-obtained adsorbents were studied by Brunauer–Emmett–Teller (BET), X-ray diffraction (XRD), X-ray photoelectron spectroscopy (XPS), Fourier transform infrared spectroscopy (FTIR), and in situ diffuse reflectance infrared Fourier transform spectroscopy (in situ-DRIFTS) to reveal the reaction mechanisms.

## 2 Experimental Section

### 2.1 Preparation of Adsorbents

Four different Cu precursors, Cu(NO_3_)_2_, CuCl_2_, Cu(C_2_H_4_O_2_)_2_, and CuSO_4_, were used for the modification of activated carbon. All adsorbents were prepared by the low-temperature roasting method. Cu(NO_3_)_2_-modified Ac was taken as an example; first, activated carbon was ground, followed by washing with deionized water and drying at 80 °C for 4 h. After sieving the particles through 40–60 mesh, 2.0 g of activated carbon was impregnated with 2.0 mol/L Cu(NO_3_)_2_ solution of 10 mL. Next, the mixtures were left to stand for 24 h after stirring at room temperature, followed by roasting in an oven at low temperatures for 5 h (80, 120, 150, and 180 °C). Afterward, the samples were cooled down to ambient temperature and sieved through 40–60 mesh. The Cu content was varied from 1 to 3 mol/L, where wt % means the mass percentage. After cooling, the CuO-modified activated carbon adsorbents were obtained. Samples prepared from different precursors are defined as Cu(NO_3_)_2_/Ac, Cu(C_2_H_4_O_2_)_2_/Ac, CuCl_2_/Ac, and CuSO_4_/Ac, respectively.

### 2.2 AsH_3_ Breakthrough Dynamic Testing

**Figure 1:**
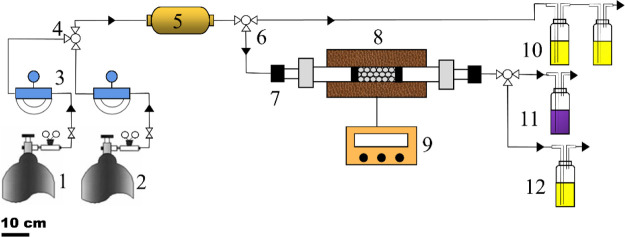
Schematic diagram of the experimental setup: (1) cylinder with AsH_3_ (200 ppm AsH_3_), (2) cylinder with O_2_, (3) mass flowmeter, (4) three-way valve, (5) mixer, (6) three-way valve, (7) heating furnace, (8) adsorbents, (9) temperature controller, (10) inlet measuring point, (11) KMnO_4_ solution for AsH_3_ tail gas adsorption, and (12) outlet measuring solution.

A scheme of the experimental setup is provided in Figure 1. The gas stream was introduced into a mixer at a gas flow rate of 200 mL/min. The gas was composed of AsH_3_ (200 ppm; Dalian Special Gas Corporation, China) diluted in O_2_ (0–1.2%). The premixed gas then entered the fixed bed filled with a 0.2 g adsorbent adsorption column. Two sample ports were set: one for the imported gas and the other for the residual tail gas after the treatment at the outlet. The waste gas was treated by acid KMnO_4_. In this study, the AsH_3_ removal efficiency and adsorption capacity were used to evaluate the arsenic capture capacity of the adsorbent. All experiments were repeated three times, and the standard deviation and average value were calculated. The reaction conditions are summarized in Table 1.

**Table 1:** Experimental Reaction Conditions

	sample				
experiment	roasting temperature	active component	carrier	reaction temperature (°C)	flue gas component
set I	180	2 mol/L Cu(NO_3_)_2_, CuCl_2_, Cu(C_2_H_4_O_2_)_2_, CuSO_4_	Ac	40	N_2_ + 1% O_2_ + 200 ppm AsH_3_
set II	80–180	2 mol/L Cu(NO_3_)_2_	Ac	40	N_2_ + 1% O_2_ + 200 ppm AsH_3_
set III	180	0–3.0 mol/L Cu(NO_3_)_2_	Ac	40	N_2_ + 1% O_2_ + 200 ppm AsH_3_
set IV	180	2 mol/L Cu(NO_3_)_2_	Ac	40	N_2_ + O_2_(0.5–1.2%) + 200 ppm AsH_3_
set V	180	2 mol/L Cu(NO_3_)_2_	Ac	25–60	N_2_ + 1% O_2_ + 200 ppm AsH_3_

The concentration of AsH_3_ was measured by the silver diethyldithiocarbamate spectrophotometry method. The concentration of AsH_3_ was calculated by eq 1 under various conditions
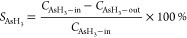
1

The adsorption capacity of AsH_3_ was calculated using eq 2
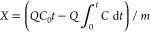
2where *X* is the adsorption capacity, mg/g; *Q* denotes the gas flow rate, m^3^/min; *t* represents the adsorption time, min; *C*_0_ and *C* represent the inlet and outlet mass concentrations of AsH_3_, respectively, mg/m^3^; *C* represents the outlet mass concentration of AsH_3_ in mg/m^3^; and *m* is the weight of the adsorbent, g.

### 2.3 Adsorbent Characterization

The surface areas of the sorbents were determined by the BET method, while the pore volume and the average pore size were extracted from the desorption branch of the isotherm and calculated by the Barrett–Joyner–Halenda method. The XRD patterns of the sorbents were recorded using a D8 Rigaku9000 X-ray powder diffractometer equipped with Ni-filtered Cu Kα radiation (λ = 0.15406 nm). The surface states of the adsorbents were analyzed by XPS (Thermo ESCALAB 250XI, Massachusetts, Waltham, USA). The Cu 2p, O 1s, and As 3d binding energies (BEs) were calibrated using C 1s (BE = 284.8 eV) as a standard. A Nicolet Impact 400 FT-IR spectrometer with a TGS detector was used for FT-IR studies. In situ-DRIFTS was carried out on a Thermo Scientific Nicolet iS50 spectrometer equipped with a mercury–cadmium–telluride detector cooled by liquid N_2_. Adsorbents were first purged at 300 °C for 2 h under N_2_ gas (a total flow rate of 100 mL/min) to dislodge water interference, and subsequently, spectra of samples at the desired temperatures were recorded.

## 3 Results and Discussion

### 3.1 Effect of Preparation Conditions on AsH_3_ Adsorption

**Figure 2:**
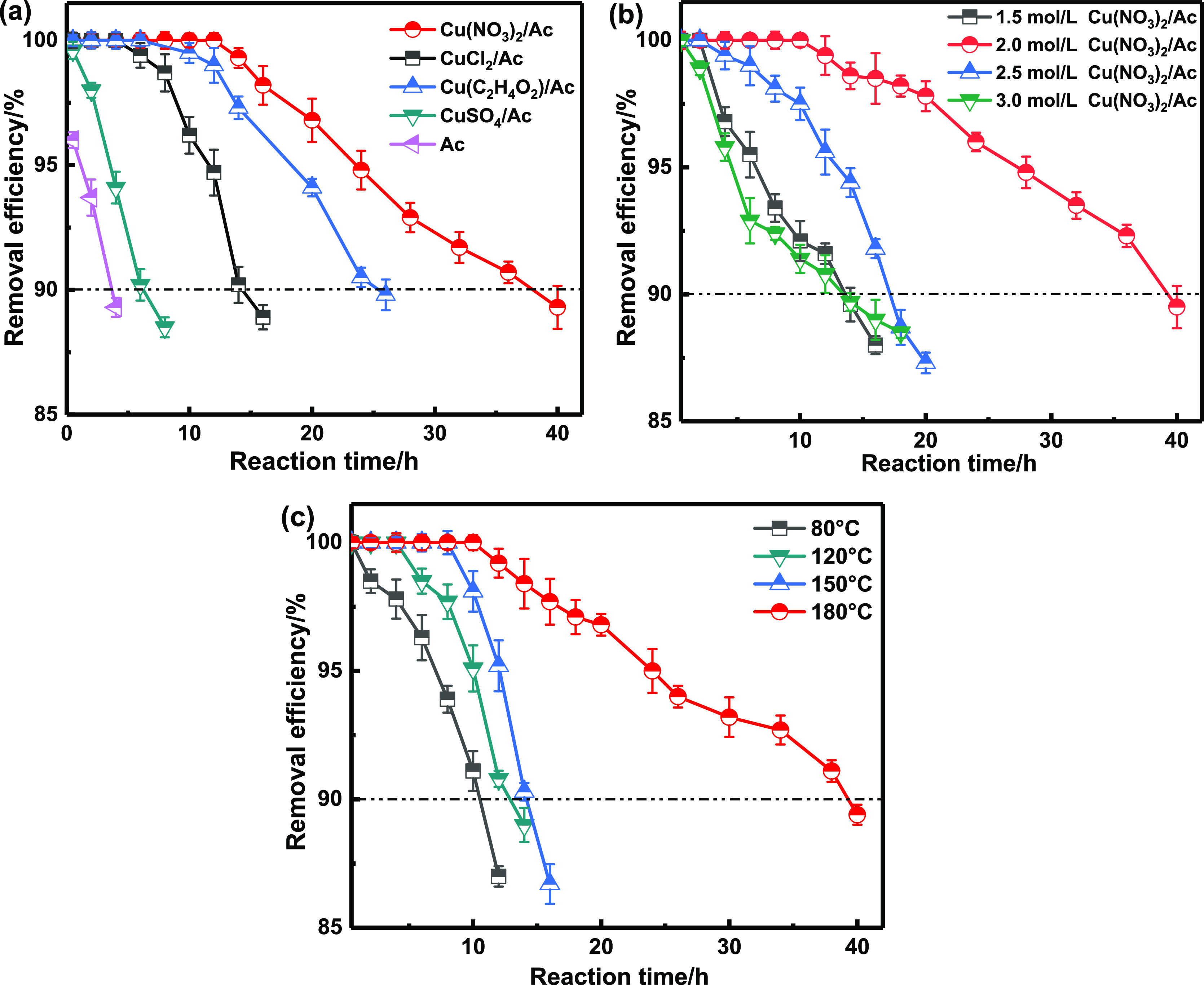
Effect of the (a) different Cu precursors, (b) Cu(NO_3_)_2_ impregnation concentration, and (c) roasting temperature on AsH_3_ removal efficiencies; Reaction conditions: reaction temperature = 40 °C, [AsH_3_] = 200 ppm, and [O_2_] = 1.0 vol %.

The breakthrough curves of AsH_3_ obtained with different Cu precursors (Cu(NO_3_)_2_, Cu(C_2_H_4_O_2_)_2_, CuCl_2_, and CuSO_4_) are depicted in Figure 2a. The breakthrough time declined in the following order: Cu(NO_3_)_2_/Ac > Cu(C_2_H_4_O_2_)_2_/Ac > CuCl_2_/Ac > CuSO_4_/Ac > Ac. The adsorbent prepared with CuSO_4_ as the precursor displayed the worst effect. After roasting at 180 °C, the removal efficiency of AsH_3_ decreased to less than 90% only 7 h. This result was slightly better than that of the blank activated carbon. The reason for this may have to do with the difficult decomposition of SO_4_^2–^ during low-temperature roasting, which prevented the formation of active components. The adsorbent prepared with CuCl_2_ and Cu(C_2_H_4_O_2_)_2_ precursors looked better than those obtained with CuSO_4_. However, the removal efficiencies of AsH_3_ were lower than 90% after 12 and 23 h. Also, the adsorbent prepared with the Cu(NO_3_)_2_ precursor was able to remove AsH_3_ for nearly 40 h. The reason for this had to do with Cu(NO_3_)_2_ precursors. Xu et al.^13^ reported the clustering tendency of Cu(NO_3_)_2_ during the impregnation process, allowing the adsorbent to retain a larger surface area and pore volume. Therefore, Cu(NO_3_)_2_ was selected as an optimal precursor in subsequent experiments for Cu modification of activated carbon. The adsorbent prepared with different active component concentrations (1.5, 2, 2.5, and 3 mol/L) is shown in Figure 2b. The adsorbent prepared with 1.5 mol/L Cu(NO_3_)_2_ illustrated poor performances toward the catalytic oxidation of AsH_3_, while that obtained with 2 mol/L Cu(NO_3_)_2_ displayed the best catalytic effect. However, the increment in loading content to 2.5 mol/L and 3 mol/L led to a decline in performance. Thus, excess Cu(NO_3_)_2_ loading not only piled up on the adsorbent surface but also blocked the pore size of the activated carbon, thereby reducing the surface active sites of the adsorbent. In turn, the performance of the adsorbent toward the catalytic removal of AsH_3_ diminished. Thus, the use of 2 mol/L Cu(NO_3_)_2_ showed the best catalytic activity. The roasting temperature would significantly impact the pore size, pore volume, and precursor oxidation products of the activated carbon adsorbent. Harsh calcination conditions used in the industrial process combined with a flue gas temperature below 200 °C are not convenient for the preparation of traditional adsorbents. As a result, adsorbents prepared without calcination would have broad prospects. The AsH_3_ reaction curves for adsorbents loaded by 2 mol/L Cu(NO_3_)_2_ and roasted at temperatures of 80, 120, 150, and 180 °C are presented in Figure 2c. The results obtained at 180 °C looked significantly improved when compared to 80, 120, and 150 °C. When roasted at 180 °C, the adsorbent displayed the best removal efficiency toward AsH_3_. This can be related to the decomposition temperature of Cu(NO_3_)_2_ (170 °C). The influences of four different roasting temperatures on materials performances will be discussed in the BET results section.

### 3.2 Characterization of the Cu/Ac Adsorbent

**Figure 3:**
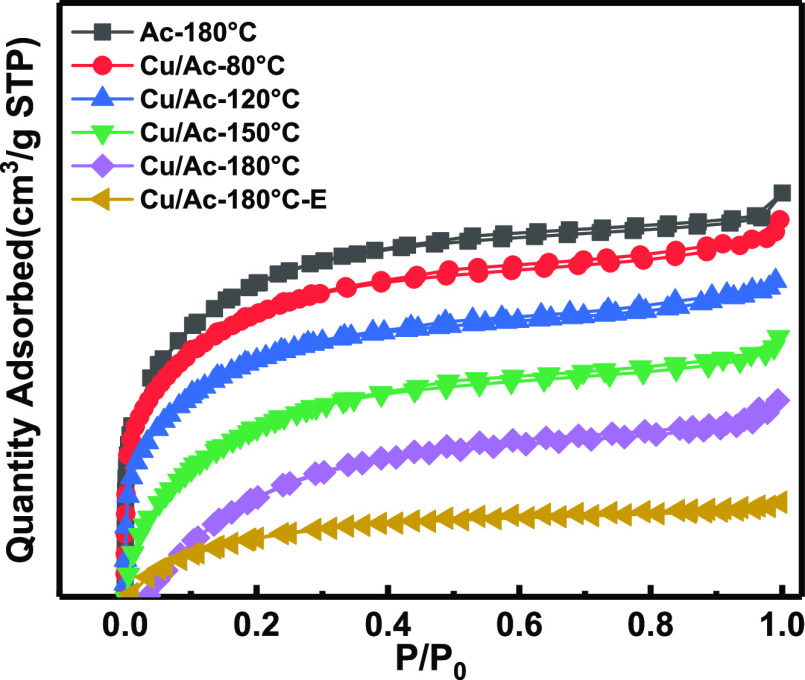
Nitrogen adsorption/desorption isotherms were obtained for blank Ac, Ac loaded with 2% copper adsorbents at different roasting temperatures, and the sample roasting at 180 °C after AsH_3_ adsorption.

The pore property of the adsorbent is the key factor to determine the adsorption performance. As shown in Figure 3, the N_2_ adsorption capacity of blank activated carbon was stronger than those of Cu/Ac-180 °C and Cu/Ac-180 °C after the reaction (denoted as Cu/Ac-180 °C-E). This can be explained by the partially covered Cu/Ac-180 °C and Cu/Ac-180 °C-E by active components as well as a decline in surface area. Moreover, the hysteresis loop shifted in the relative pressure range of 0.4–1.0, a typical characteristic of mesoporous structures. The physical properties of adsorbents prepared with 2% Cu(NO_3_)_2_ at different roasting temperatures of 80, 120, 150, and 180 °C are gathered in Table 2. This included BET surface area, total pore volume, DFT pore size, and average pore size. The BET surface area of blank activated carbon decreased after loading, while the surface area reached the maximum 1004.98 m^2^/g at 180 °C roasting. These data were consistent with those of the removal effect, confirming that the best roasting temperature is 180 °C. It was speculated that copper nitrate was not completely decomposed at the other roasting temperatures, so this did not form the copper oxide crystal phase, that is, not enough active sites. Therefore, the following experiments could be carried out on samples roasting at 180 °C (denoted as Cu/Ac). In addition, it was observed that the specific surface area sharply decreased to 427.78 m^2^/g after AsH_3_ adsorption, accompanied by a reduction to 0.22 cm^3^/g in pore volume, which may be due to deposition of adsorption products on the surface of the Cu/Ac adsorbent.

**Table 2:** Specific Surface Area, Total Pore Volume, DFT Pore Size and Average Pore Size of Blank Ac, Ac Loaded with 2% Copper Adsorbents at Different Roasting Temperatures, and the Sample Roasting at 180 °C after AsH_3_ Adsorption

sample	BET surface area (m^2^/g)	total pore volume (cm^3^/g)	DFT pore size (D/nm)	average pore size (D/nm)
Ac-180 °C	1094.35	0.54	0.56	1.96
Cu/Ac-80 °C	937.87	0.50	1.13	2.14
Cu/Ac-120 °C	886.80	0.47	1.13	2.10
Cu/C-150 °C	963.21	0.51	1.03	2.12
Cu/C-180 °C	1004.98	0.53	0.56	2.12
Cu/C-180 °C-E	427.78	0.22	0.88	2.04

**Figure 4:**
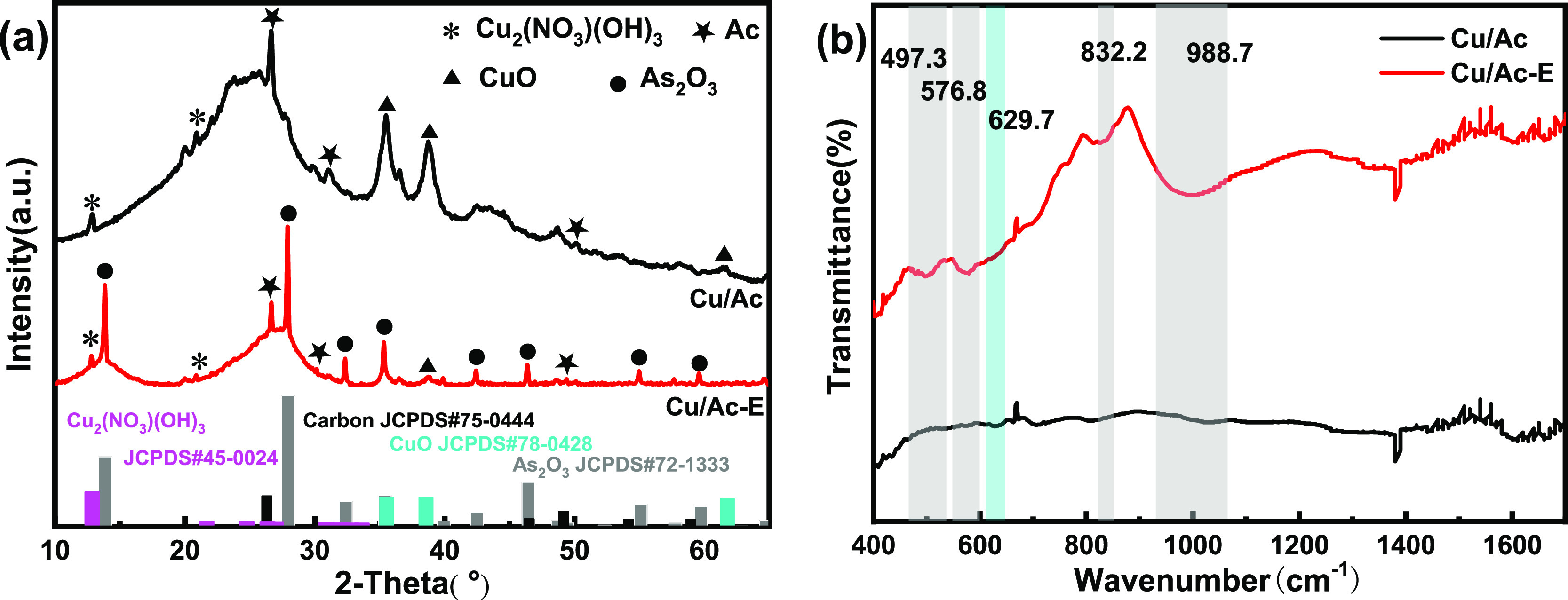
XRD patterns (a) and FTIR results (b) of Cu/Ac and Cu/Ac-E.

XRD and FTIR characterization were used to further determine the deposition products on the adsorbent and the changes of the surface phase after reaction. Figure 4a shows the XRD patterns of the prepared sample with 2% copper content. For the Cu/Ac sample, characteristic peaks of CuO were present at 35.6°, 38.8°, and 61.8°. CuO was successfully deposited on the adsorbent surface as an active component. In addition, the Cu/Ac sample exhibited some diffraction peaks at 12.8°,^14,15^ corresponding to cupric nitrate (Cu_2_(NO_3_)(OH)_3_). Therefore, the first consisted of transforming into Cu_2_(NO_3_)(OH)_3_, and the second consisted of transforming into copper oxide. The weak interaction between copper atoms and activated carbon led to the facile reduction of copper ions to Cu^+^, thereby promoting the reaction. Cu(NO_3_)_2_ clusters occurred during impregnation so that the adsorbent retained a larger surface area and pore volume, which is consistent with the BET analysis. In addition, diffraction peaks of As_2_O_3_ were detected at 28.0°, 32.2°, 35.5°, 42.6°, 46.4°, 55.0°, and 59.6° in the Cu/Ac-E sample.^16^ Meanwhile, the peak attributed to trace copper may be overlaid by that of As_2_O_3_. However, the relevant diffraction peak of As_2_O_5_ was absent in the XRD spectrum of related adsorbents obtained after the reaction. This may be caused by the low content of As_2_O_5_ present on the adsorbent surface. Note that the existence of As_2_O_5_ was confirmed by subsequent characterizations. Moreover, the background spectrum of the activated carbon remained almost unchanged after reaction, indicating the presentation of the carrier structure of the modified activated carbon as well as the low effect of the arsenic oxide product on the pore structure of the carrier. The IR spectra of Cu/Ac and Cu/Ac-E are shown in Figure 4b. Two new peaks appeared at 497.3 and 576.8 cm^–1^ of Cu/Ac-E, which were related to the presence of O–As–O.^17^ Another two new peaks at 832.2 and 988.7 cm^–1^ were associated with the formation of As–O after the reaction.^18^ During the adsorption process, some reactions of active sites took place. After the adsorption of AsH_3_ on the CuO surface, the bands at 629.7 cm^–1^ weakened due to the bond cleavage reactions of Cu–O.^19^ The exposure to AsH_3_ resulted in the deposition of a significant quantity of arsenic on the surface as well as an increased percentage of oxygen for the CuO series.^17^ These results further testified that CuO was the active center for AsH_3_ removal.

**Figure 5:**
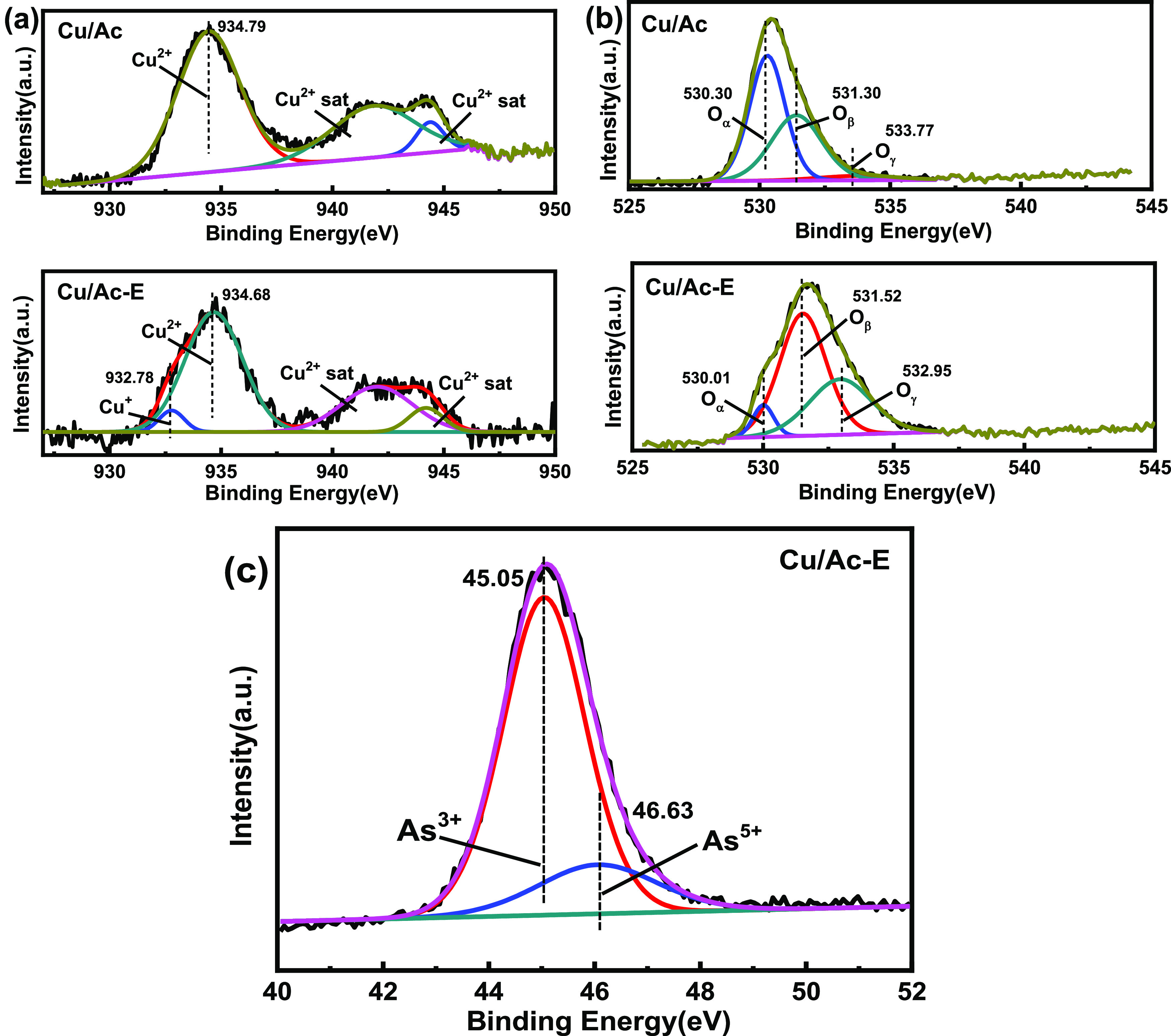
XPS spectra of Cu 2p (a), O 1s (b), and As 3d (c) for the Cu/Ac and Cu/Ac-E adsorbents.

To investigate the surface species and chemical states of O 1s, Cu 2p, and As 3d in the as-prepared adsorbents, XPS was employed for further characterization of the samples, and the data are summarized in Figure 5. The corresponding XPS data before and after the reaction are displayed in Table 3. The BEs in the Cu 2p spectra are depicted in Figure 5a. The Cu 2p peaks centered at 934.68–934.79 eV were assigned to the Cu^2+^ in the Cu/Ac and Cu/Ac-E samples.^12,20^ The Cu^2+^ content of the adsorbent decreased from 100.00 to 75.35% after the reaction, with the appearance of a peak of Cu^+^ at 932.8 eV. Considering the conversion cycle of Cu^2+^ and Cu^+^ contributing to oxygen utilization, the involvement of Cu species on the surface of Cu/Ac plays an important role in enhancement of AsH_3_ removal.^21^ Three peaks of O 1s are observed in Figure 5b. The peaks at about 531.3–532.3 eV could be attributed to chemisorbed oxygen (denoted as O_β_). By comparison, those at 530.01–530.30 eV belonged to lattice oxygen (donated as O_α_), and those at 534.00–534.50 eV belonged to hydroxyl oxygen or adsorbed molecular water (denoted as O_γ_).^22−24^ The O_α_ decreased after the reaction, while the O_β_ increased by 17.34%. During the catalytic reaction, the conversion cycle of Cu^2+^ and Cu^+^ can consume O_α_, and oxygen in the air was absorbed on the surface of the adsorbent to supplement O_β_. However, after the interaction of O_β_ with ions on the surface, it was converted into O_α_ to obtain electrons, thus achieving dynamic equilibrium. When oxygen supply was insufficient during the reaction, O_α_ in the active component CuO will be converted to O_α_ to participate in the reaction of AsH_3_. This is consistent with the reduction of Cu^2+^ after the reaction in the previous analysis. In Figure 5c, the As 3d spectra of reaction samples were divided into two peaks. The one located at 46.63 eV was attributed to As_2_O_3_ (81.77%), while the other located at 45.05 eV was ascribed to As_2_O_5_ (18.23%). In other words, As_2_O_3_ was the dominant oxidation product in the reaction.^21^

**Table 3:** Relative Contents of Surface Species of Cu/Ac and Cu/Ac-E^a^

	atomic concentration (at. %)						
sample	Cu^+^	Cu^2+^	O_α_	O_β_	O_γ_	As^3+^	As^5+^
Cu/Ac	—	100	54.83	40.80	4.37	—	—
Cu/Ac-E	24.65	75.35	6.79	58.14	35.06	81.77	18.23

a Annotation: the symbol “—” represents a value of 0.

### 3.3 Effect of Reaction Conditions on AsH_3_ Adsorption over Cu/Ac

**Figure 6:**
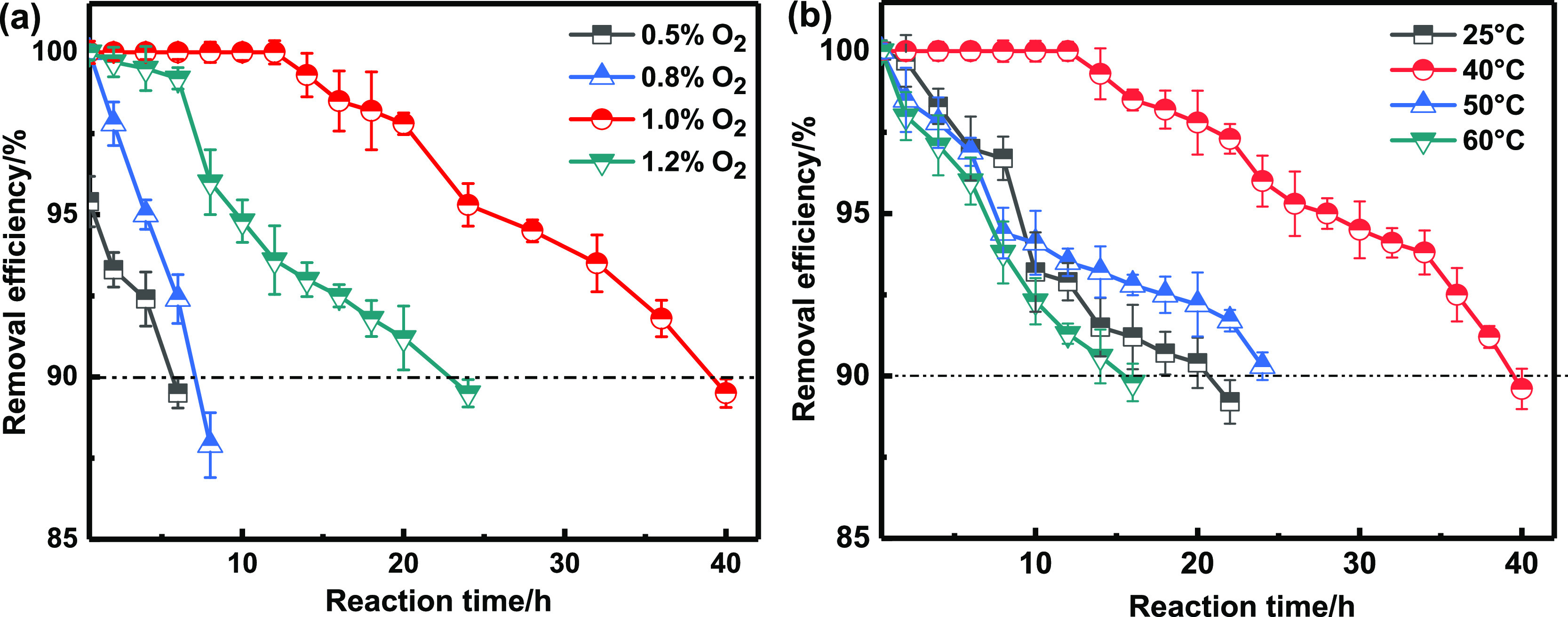
Effect of the oxygen content (a) and reaction temperature (b) on AsH_3_ removal efficiency. Reaction conditions: Cu(NO_3_)_2_ content = 2 mol/L, roasting temperature = 180 °C, [AsH_3_] = 200 ppm.

The adsorption performance of Cu/Ac under actual industrial conditions of micro-oxygen and low temperature was tested. As shown in Figure 6a, the removal efficiency of AsH_3_ increased with oxygen content (0.5, 0.8, 1, and 1.2%). The removal efficiencies registered at oxygen contents of 0.5 and 0.8% were lower than that recorded at 90% after 8 h. By comparison, a significant increase was obtained with a 1% oxygen content. However, a further rise in oxygen content to 1.2% declined the performance since the presence of too much oxygen would compete with AsH_3_ for active sites. Therefore, 1.0% oxygen content was selected as optimal and used in further reaction. The low-temperature adsorption performance of Cu/Ac was verified at 25, 40, 50, and 60 °C, and the results are illustrated in Figure 6b. At 25 °C, the initial removal efficiency of AsH_3_ was low but improved at 40 °C. This could be due to the activation temperature of the adsorbent. However, a further rise in temperature to 50 and 60 °C led to a decline in removal efficiency to yield values about the same as those obtained at 25 °C. Therefore, 40 °C was selected as the optimal reaction temperature to yield suitable adsorbents. The adsorption capacity reached 369 mg/g under these conditions, which was 10 times that of the other copper-containing adsorbent. The above result proved that Cu/AC had excellent arsenic removal capacity under industrial micro-oxygen and low-temperature conditions.

**Figure 7:**
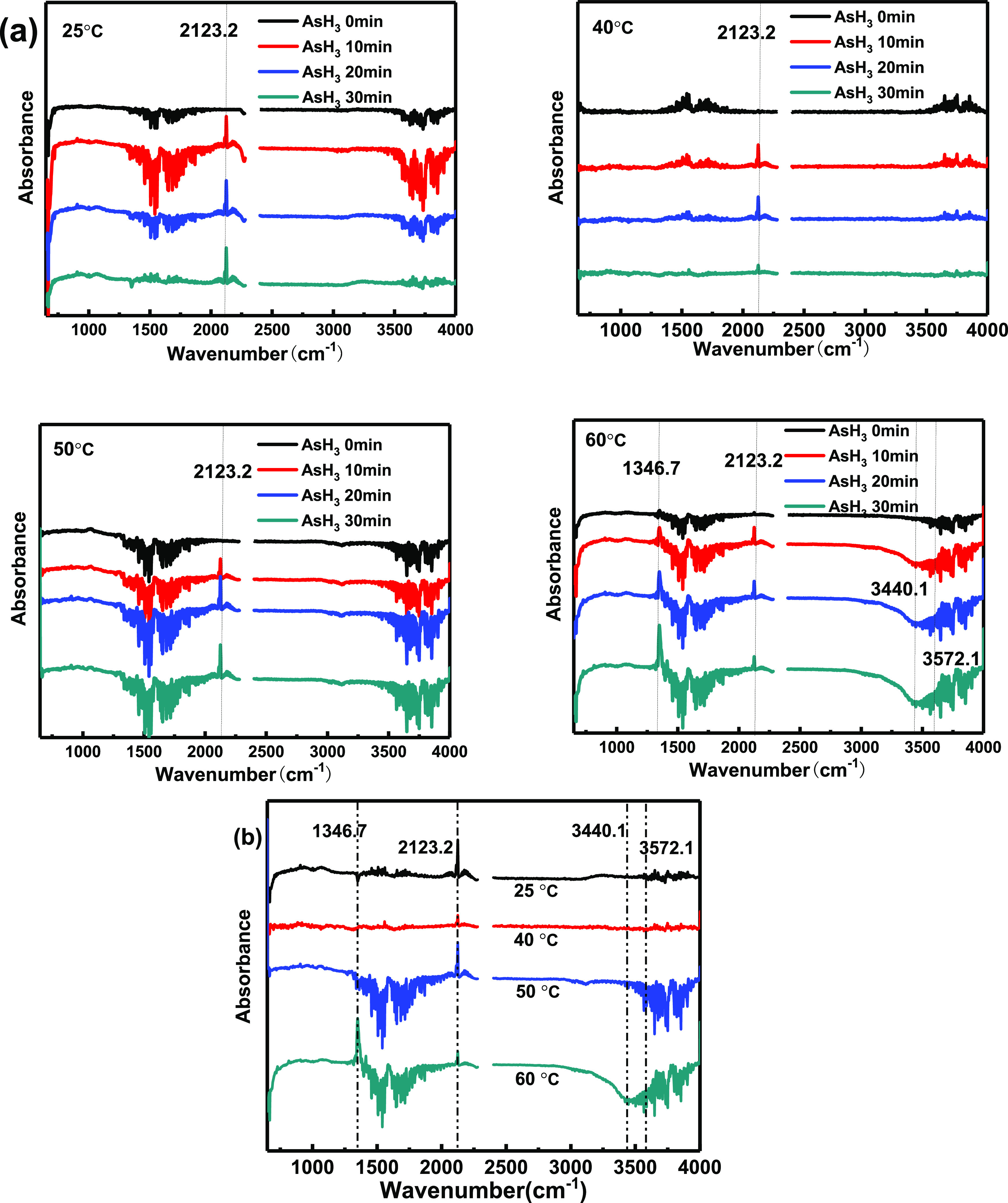
(a) Reaction curve obtained every 10 min after passing AsH_3_ in 25, 40, 50, and 60 °C. (b) In situ DRIFTS obtained after 30 min AsH_3_ adsorption.

Cu/Ac was further characterized by in situ DRIFTS at different reaction temperatures to investigate its excellent properties at low temperature. The reaction curves were taken every 10 min after passing AsH_3_ at 25, 40, 50, and 60 °C and are displayed in Figure 7a within the range of 650–4000 cm^–1^. The comprehensive spectra of after passing AsH_3_ for 30 min are provided in Figure 7b. All the curves showed the AsH_3_ characteristic peak at 2123.2 cm^–1^,^25^ and the intensity changes were different at different temperatures. The intensity was the highest at 25 °C, while it was the lowest at 40 °C. Based on an obvious abatement of the AsH_3_ characteristic peak along with the time increasing in Figure 7a, it can be inferred that the Cu/Ac sample has a good purification capacity on AsH_3_ at 40 °C, which was consistent with the experimental phenomenon. By further comparing the curves, it was only when the reaction temperature was 60 °C that new peaks appeared at 1346.7 cm^–1^ and could be inferred to NO_3_^–^ that originates from (Cu(NO_3_)_2_) used in the adsorbent preparation process,^26−28^ which was the B acid site of the Cu/Ac adsorbent itself.^29,30^ Besides, the stretching vibration absorption peaks of the symmetrical H–O–H group appeared at 3440.1 cm^–1^,^31^ indicating that there may be catalytic reaction of AsH_3_ at the B acid site on the sample surface.^32^ However, the negative peak of 3572 cm^–1^ attributed to the OH stretching vibration of the Si–(OH)–Al bridge was observed due to toxic effects of arsenic species on B acid sites. In other words, it is precisely the toxic effect of arsenic species on strong acid sites that leads to the unsatisfactory removal efficiency when the catalytic activation temperature is reached.^33^

**Figure 8:**
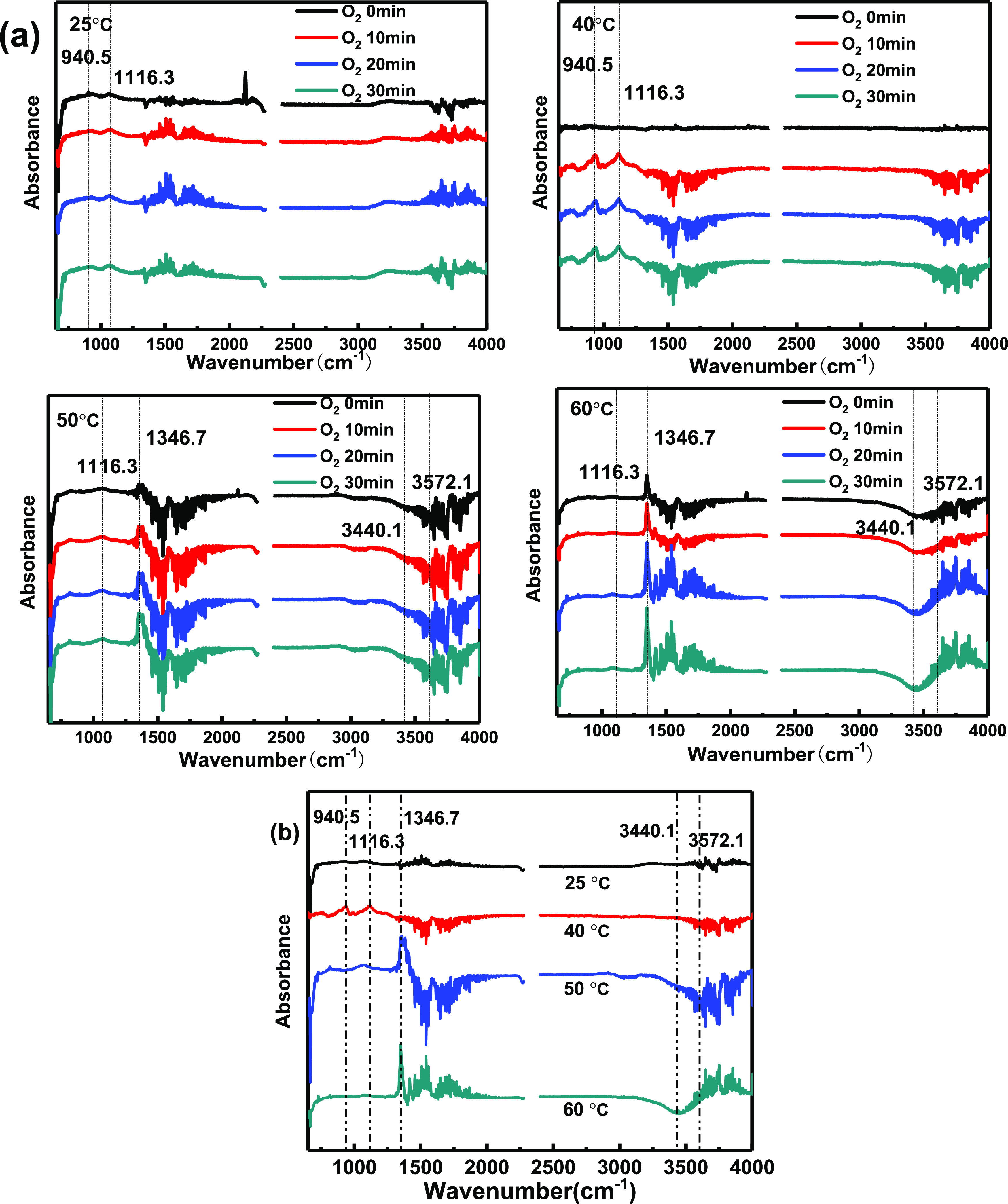
(a) Reaction curves obtained every 10 min after passing O_2_ in 25, 40, 50, and 60 °C. (b) In situ DRIFTS after 30 min adsorption of O_2_.

The reaction curves obtained every 10 min after passing O_2_ are displayed in Figure 8a, and the comprehensive spectra after passing oxygen for 30 min are illustrated in Figure 8b. When the reaction temperature was 50 and 60 °C, there was a strong peak at 1346.7 cm^–1^, along with the band at 3440.1 cm^–1^ assigned to the H–O–H group. Moreover, the negative peak of the OH stretching vibration of the Si–(OH)–Al bridge at 3572.1 cm^–1^^34^ proved again that the B acid site was involved in the catalytic reaction. Compared to the results obtained without O_2_ in Figure 7, the intensity of these peaks enhanced. The catalytic reaction took place between AsH_3_ and O_2_ at 50 and 60 °C. Two new peaks appeared at 940.5 and 1116.3 cm^–1^ at 25 and 40 °C; the former was assigned to the presence of As–O from the arsenic oxide product,^35^ and the latter was assigned to the bonds of the Lewis (L) acid sites.^36^ O_2_ may participate in the chemical adsorption of hydrogen arsenide by adsorbing at L acid sites on the surface of Cu/Ac to convert to O_β_.^33^ This indicated that a chemical adsorption process mainly occurred on the Cu/AC surface when the reaction temperature was 25 and 40 °C. Finally, the weak peak vibration of the L acid site was also observed in the reaction curve at 50 °C, concluding that chemical adsorption and catalytic oxidation processes happened at the same time. The above results showed that when the reaction temperature falls short of the catalytic activation temperature, there is mainly a chemical adsorption process on the Cu/Ac surface; however, when it is reached, catalytic oxidation is dominant. The different changes of these surface functional groups were consistent with the AsH_3_ removal efficiency at four reaction temperatures.

### 3.4 Regeneration of the Cu/Ac Adsorbent

**Figure 9:**
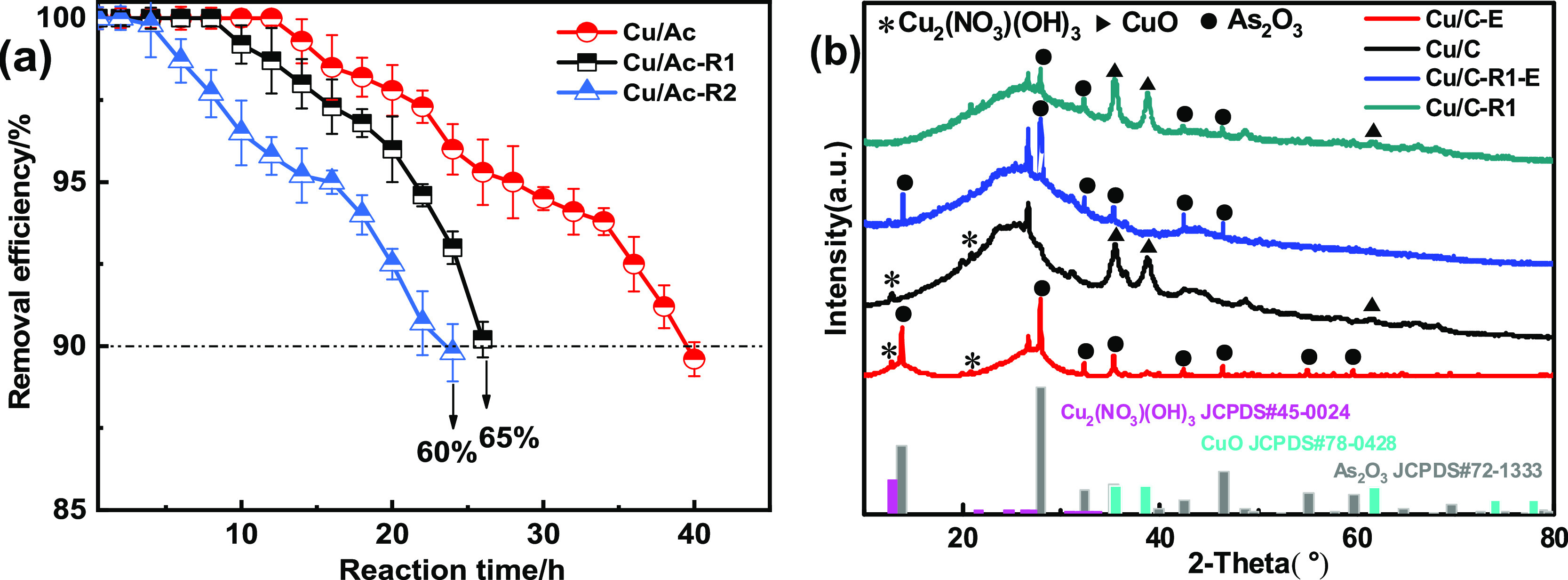
(a) Regeneration performance of Cu/Ac (reaction conditions: reaction temperature = 40 °C, [AsH_3_] = 200 ppm, and [O_2_] = 1.0 vol %); (b) XRD patterns of Cu/Ac, Cu/Ac-E, Cu/Ac-R1, and Cu/Ac-R1-E.

**Figure 10:**
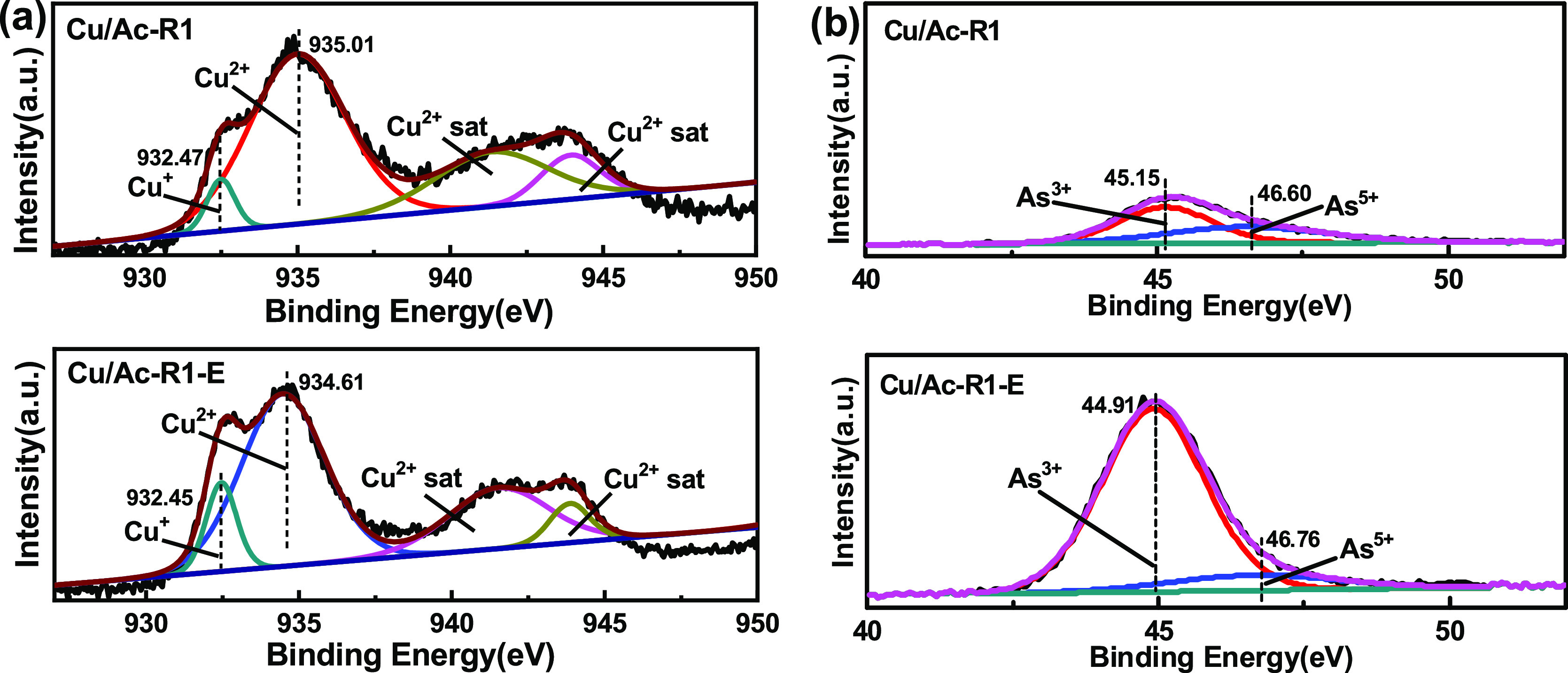
XPS spectra of Cu 2p (a) and As 3d (b) for Cu/Ac-R1 and Cu/Ac-R1-E.

The Cu/Ac adsorbent was regenerated by water washing and the purging gas method. To this end, the Cu/Ac-E sample was first activated by hot air at 100 °C for 3 h. Then, it was soaked and cleaned with deionized water four to five times and placed in an electric blast roasting oven to dry at 110–120 °C. Afterward, the sample was placed in a tubular furnace under a nitrogen atmosphere, and the temperature was increased using a temperature-programmed controller. Considering the material obtained by roasting at 180 °C, the temperature was first set at 20–180 °C. Afterward, the heating was stopped, and the sample was cooled down to room temperature. The sample was taken out, the nitrogen flow was stopped, and the regenerated material was tested. As shown in Figure 9a, the regeneration efficiency of *R* (*R* is the ratio of the breakthrough time of the regenerated adsorbent and fresh adsorbent) after one regeneration cycle was 65%. After the second regeneration cycle, the sample recovered an efficiency *R* reaching 60%, confirming reasonable regeneration capability of the Cu/Ac adsorbent toward the removal of AsH_3_. To determine the change of the surface phase after regeneration of the Cu/AC adsorbent, Cu/Ac-R1 and Cu/Ac-R1-E were analyzed by XRD. As shown in Figure 9b, after regeneration, it was evident that the intensity of the diffraction peak of CuO at 35.6°, 38.8°, and 61.8° in Cu/Ac-R1 increased, but no peak of Cu_2_(NO_3_) (OH)_3_ was found. This indicated that some CuO species recovered in the regeneration process may be transformed by intermediate Cu_2_(NO_3_)(OH)_3_. In addition, the crystallization peak of As_2_O_3_ at 28.0°, 32.2°, 35.5°, 42.6°, 46.4°, 55.0°, and 59.6° was significantly weakened in the Cu/Ac-R1 sample. It was speculated that transformation of the intermediate Cu_2_(NO_3_)(OH)_3_ to the adsorption active center CuO and separation of crystalline As_2_O_3_ may be the reasons for the good regeneration performance of the Cu/Ac adsorbent. The results of XPS further confirmed this point. The XPS results of Cu/Ac-R1 and Cu/Ac-R1-E further confirmed this point. In Figure 10a, the Cu 2p diffraction peak at 934.61 and 935.01 eV belonged to Cu^2+^ species, while the diffraction peak at 932.45 and 932.47 eV was ascribed to Cu^+^ species. These Cu^2+^ and Cu^+^ species appear in the form of CuO and Cu_2_O, respectively. From Table 4, after the reaction of Cu/Ac-R1, the content of Cu^+^ increased from 4.57 to 8.74% along with a decrease in Cu^2+^, indicating that recovered active center CuO participated in a new round of AsH_3_ adsorption. The XPS spectrum of As 3d of Cu/Ac-R1 and the Cu/Ac-R1-E sample was divided into two kind of peaks, as shown in Figure 10b. One was the characteristic peak of As^3+^ at 44.91 and 45.15 eV, and the other was of As^5+^ centered in 46.60 and 46.76 eV. It can be seen that the peak area of arsenic oxide products in the Cu/Ac-R1 sample was greatly reduced, especially a decrease in area of about 7 times of As^3+^. However, the content of As^3+^ increased from 56.27 to 87.8% in Cu/Ac-R1-E. Due to more toxicity than As_2_O_5_, As_2_O_3_ can be considered as the main reason for the deactivation of the Cu/Ac adsorbent.

**Table 4:** Relative Contents of Surface Species of Cu/Ac-R1 and Cu/Ac-R1-E

	atomic concentration (at. %)			
sample	Cu^+^	Cu^2+^	As^3+^	As^5+^
Cu/Ac-R1	4.57	95.43	56.27	43.73
Cu/Ac-R1-E	8.74	91.26	87.80	12.20

### 3.5 Identification of the AsH_3_ Adsorption Mechanism

According to the experimental results and subsequent product analysis, the mechanism of AsH_3_ chemisorption under low temperature over Cu/Ac was identified. Addition of Cu(NO_3_)_2_ to the activated carbon can form the highly active phase of CuO upon roasting at 180 °C. Since AsH_3_ was adsorbed on the metal component, the As–H bond weakened, making it easy to break at a lower temperature, and the Cu^2+^ and Cu^+^ conversion cycle of the Cu/Ac adsorbent played an important role in this process. Released O_α_ oxidized AsH_3_ to As^3+^, while Cu^2+^ was restored to Cu^+^ and then oxidized to Cu^2+^ after contacting with the oxygen of the air. Meanwhile, under a 40 °C reaction temperature, L acid sites could greatly improve the surface chemical adsorption of O_2_ of the Cu/Ac adsorbent, resulting in the supplement of O_β_ to contribute to the circulation of Cu. The main arsenic oxide products were mainly As_2_O_3_ and a small amount of As_2_O_5_. In general, the Cu/Ac adsorbent realized the efficient capture and purification of AsH_3_ through the synergic effect of the circulation of Cu species and activated L acid site.

## 4 Conclusions

The Cu/Ac adsorbent synthesized by the low-temperature roasting method was proved to have a high efficiency in capturing AsH_3_. The released O_α_ through the cycle of Cu^2+^ to Cu^+^ in the active component CuO played an essential role in the oxidation process of AsH_3_. Moreover, the L acid site of the Cu/Ac adsorbent could quickly improve the chemisorption of O_2_, thus supplementing O_β_ to assist with the cycle of Cu species. AsH_3_ was first oxidized to As_2_O_3_ and then to As_2_O_5_, leading to the formation of deposits on the adsorbent surface. As for the regeneration efficiency of 60% after several generations, the intermediate Cu_2_(NO_3_)(OH) in Cu/Ac could be partially converted into the active center CuO during the regeneration process. The results of one-time water washing and purging gas regeneration showed that Cu^2+^ was restored by 20%, while the main poison As_2_O_3_ was reduced approximately 7 times. Compared with traditional copper adsorbents, Cu(NO_3_)_2_-modified Ac roasting at low temperature reduced the reaction operating temperature by 80 °C. In terms of industrial actual conditions and low operating temperatures, Cu/AC synthesized by the low-temperature roasting method is a promising adsorbent for industrial low-temperature AsH_3_ removal.
